# P32 Real-world evaluation of a rapid point-of-care urine analyser Sysmex PA-100: implications for antimicrobial stewardship

**DOI:** 10.1093/jacamr/dlag102.038

**Published:** 2026-06-26

**Authors:** Niamh M Murphy, Mia Outteridge, Megan E Russell, Richard M Dawood

**Affiliations:** Fleet Street Clinic, London, UK; Fleet Street Clinic, London, UK; Fleet Street Clinic, London, UK; Fleet Street Clinic, London, UK

## Abstract

**Objectives:**

Rapid point-of-care (POCT) diagnostics for urinary tract infection (UTI) aim to support timely antimicrobial prescribing and improve antimicrobial stewardship. The Sysmex PA-100 provides bacteriuria detection and phenotypic antimicrobial susceptibility testing (AST) within 45 minutes. Published real-world performance data remain limited. This study aimed to evaluate the clinical performance and utility of the PA-100 in an outpatient setting.

**Methods:**

A service evaluation of 22 urine samples in a London general practice clinic was performed. Samples included both in-scope and out-of-scope cases relative to the analyser’s intended use. PA-100 bacteriuria and AST results were compared with the referral laboratory's urine culture and susceptibility testing results.

**Results:**

Sensitivity for bacteriuria detection was 64%. One discordant positive result was identified; however, delayed culture processing limited interpretation. False negatives occurred across both in-scope and out-of-scope samples, consistent with the manufacturer’s guidance regarding organism range and detection thresholds. AST results showed partial concordance with reference testing. In positive samples, the analyser frequently identified resistance to key antibiotics (e.g. co-amoxiclav, trimethoprim) and suggested clinically appropriate alternatives (e.g. fosfomycin, nitrofurantoin). Discrepancies were observed, particularly in mixed infections and Gram-positive organisms. In some cases, results supported clinical decisions to withhold or refine empirical antibiotic therapy.

**Conclusions:**

While negative results do not reliably exclude UTI and culture remains required, positive results may support antimicrobial stewardship by enabling early exclusion of inappropriate empirical therapies and identification of suitable first-line options. These findings highlight both the potential and limitations of rapid POCT urine diagnostics in real-world clinical practice.

